# What Is Cod-Liver Oil?

**Published:** 1867-03

**Authors:** 


### *What is Cod-Liver Oil?*

The Bridgewater Gazette, a New England journal, says that a physician in that place was recently called to prescribe for a somewhat illiterate old lady, and as cod-liver oil, in his opinion, was the remedy for her complaint, he wrote a prescription for the apothecary to put up, with the Latin formula, "Ol. Jec. Ass.," being an abbreviation of "oleum jecoris asselli," or in plain English, cod-liver oil. The medicine was procured, taken, and in a few weeks the lady completely recovered her health. A neighbor paid her a visit after her recovery, and, expressing surprise at her improved condition, inquired the secret of so rapid a restoration. "Why," said the old lady, lifting both hands in grateful enthusiasm, "it was that *beautiful* medicine, the *oil of Jackass*, that brought me on my feet again!"

